# Blood Cell Mitochondrial DNA Content and Premature Ovarian Aging

**DOI:** 10.1371/journal.pone.0042423

**Published:** 2012-08-03

**Authors:** Marco Bonomi, Edgardo Somigliana, Chiara Cacciatore, Marta Busnelli, Raffaella Rossetti, Silvia Bonetti, Alessio Paffoni, Daniela Mari, Guido Ragni, Luca Persani, the Italian Network for the study of Ovarian Dysfunctions, M. Arosio, P. Beck-Peccoz, M. Biondi, S. Bione, V. Bruni, C. Brigante, S. Cannavo`, L. Cavallo, M. Cisternino, I. Colombo, S. Corbetta, P.G. Crosignani, M.G. D'Avanzo, L. Dalpra, C. Danesino, E. Di Battista, F. Di Prospero, E. Donti, S. Einaudi, A. Falorni, C. Foresta, F. Fusi, N. Garofalo, I. Giotti, R. Lanzi, D. Larizza, N. Locatelli, P. Loli, S. Madaschi, M. Maghnie, S. Maiore, F. Mantero, A. Marozzi, S. Marzotti, N. Migone, R. Nappi, D. Palli, M.G. Patricelli, C. Pisani, P. Prontera, F. Petraglia, G. Radetti, A. Renieri, I. Ricca, A. Ripamonti, R. Rossetti, G. Russo, S. Russo, M. Tonacchera, D. Toniolo, F. Torricelli, W. Vegetti, N. Villa, P. Vineis, M. Wasniewsk, O. Zuffardi

**Affiliations:** Milan; Milan; Avellino; Pavia; Florence; Milan; Messina; Bari; Pavia; Milan; Milan; Milan; Avellino; Monza; Pavia; Genova; Civitanova Marche-AN; Perugia; Torino; Perugia; Padova; Milan; Palermo; Florence; Milan; Pavia; Milan; Milano; Milan; Genova; Roma; Padova; Milan; Perugia; Turin; Pavia; Florence; Milan; Pavia; Perugia; Siena; Bolzano; Siena; Pavia; Milan; Milan; Milan; Varese; Pisa; Milano; Florence; Milan; Monza; London; Messina; Pavia; 1 Laboratori di Ricerche Endocrino-Metaboliche e, Fondazione Istituto Di Ricovero e Cura a Carattere Scientifico Istituto Auxologico Italiano, Milan, Italy; 2 UO di Medicina Generale ad indirizzo Endocrino-Metabolico, Fondazione Istituto Di Ricovero e Cura a Carattere Scientifico Istituto Auxologico Italiano, Milan, Italy; 3 Centro Sterilità, Fondazione Istituto Di Ricovero e Cura a Carattere Scientifico Ca’ Granda Ospedale Maggiore Policlinico, Milan, Italy; 4 Dipartimento di Scienze Cliniche e di Comunità, Università degli Studi di Milano, Milan, Italy; Charité Universitätsmedizin Berlin, NeuroCure Clinical Research Center, Germany

## Abstract

Primary ovarian insufficiency (POI) is a critical fertility defect characterized by an anticipated and silent impairment of the follicular reserve, but its pathogenesis is largely unexplained. The frequent maternal inheritance of POI together with a remarkable dependence of ovarian folliculogenesis upon mitochondrial biogenesis and bioenergetics suggested the possible involvement of a generalized mitochondrial defect. Here, we verified the existence of a significant correlation between blood and ovarian mitochondrial DNA (mtDNA) content in a group of women undergoing ovarian hyperstimulation (OH), and then aimed to verify whether mtDNA content was significantly altered in the blood cells of POI women. We recruited 101 women with an impaired ovarian reserve: 59 women with premature ovarian failure (POF) and 42 poor responders (PR) to OH. A Taqman copy number assay revealed a significant mtDNA depletion (*P*<0.001) in both POF and PR women in comparison with 43 women of similar age and intact ovarian reserve, or 53 very old women with a previous physiological menopause. No pathogenic variations in the mitochondrial DNA polymerase γ (*POLG*) gene were detected in 57 POF or PR women with low blood mtDNA content. In conclusion, blood cell mtDNA depletion is a frequent finding among women with premature ovarian aging, suggesting that a still undetermined but generalized mitochondrial defect may frequently predispose to POI which could then be considered a form of anticipated aging in which the ovarian defect may represent the first manifestation. The determination of mtDNA content in blood may become an useful tool for the POI risk prediction.

## Introduction

Primary ovarian insufficiency (POI) is a critical fertility defect characterized by an anticipated impairment of the follicular reserve [1], [2]. This process is generally silent without evident menstrual irregularity so that women are diagnosed with POI due to the premature cessation of menses (secondary amenorrhea, SA) before 40 years of age in the stage of complete follicular depletion (premature ovarian failure, POF or overt POI).

The cause of POI remains obscure in most women [3], [4]. A strong genetic component in idiopathic POI pathogenesis is revealed by the frequent familiarity (30–40% of total cases) for this disease [5], with mothers and daughters experiencing a premature ovarian aging and an anticipation of menopause. Among the familial cases, the maternal inheritance of the ovarian defect is prevalent (about 60%) [5], [6]. This observation together with the known association of POF with X-chromosomal abnormalities have prompted studies on the possible involvement of X-linked genes in POI pathogenesis [4], [7], [8]. However, the mitochondrial material of the zygote has a maternal origin and also mitochondrial diseases may have a maternal transmission. Noteworthy, mitochondrial defects are strongly associated with physiological or pathological tissue aging, and can lead to altered metabolism, impaired activity and/or accelerated degeneration [9], [10].

Mitochondrial biogenesis and bioenergetics play an essential role in oocyte maturation and embryo development [11], [12]. Mature oocytes require one of the highest number of mitochondria among all tissues in the body, and mitochondrial DNA copy number per oocyte has been shown to be strictly associated to the probability to develop a zygote [13], [14]. In line with these findings, intracytoplasmatic transfer of mitochondria markedly improves the chances of pregnancy [12], [13]. In addition, a marked mitochondrial depletion in the oocyte has been observed in women with poor recoveries of fertilizable oocytes after ovarian hyperstimulation [15] and, importantly, also in cases of ovarian insufficiency [16]. In addition, it has been reported that granulosa cells from women above 38 years of age have a diminished number of mitochondria when compared to those from younger fertile women [17]. The whole of these data indicate that a reduction in the number of intact mitochondria correlates with ovarian aging thus suggesting that a weakened respiratory chain activity may be involved in the anticipated impairment of ovarian reserve.

A correlation between mitochondrial diseases and POI has been observed both in animals and in humans [18]–[21]. In particular, germline mutations in the mitochondrial DNA (mtDNA) polymerase-γ (*POLG;* MIM 174763) gene cause a generalized mtDNA replication impairment and mitochondrial depletion and are associated with phenotypes variably including neurological and muscular defects, diabetes and POI.

The aim of the present study was to verify whether the content of mtDNA is significantly reduced in the blood cells of women with a prematurely impaired ovarian reserve. Therefore, we verified the possible existence of a correlation between blood and ovarian mtDNA content and then evaluated mtDNA content in peripheral blood cells of women with POF or an anticipated impairment of their ovarian reserve and in two control groups: one constituted by women with intact ovarian reserve and the second by very old women reporting a physiological menopause. Since responsiveness to ovarian hyper-stimulation is currently believed to be the most appropriate surrogate way to assess ovarian reserve [22], women belonging to the impaired and intact ovarian reserve groups were recruited among patients undergoing *in vitro* fertilization (IVF) cycles.

## Materials and Methods

Approval for the study was obtained by the local Institution review board and all subjects gave their informed consent for granulosa cells (GCs) and/or blood sampling and genetic analysis.

### Subjects

In a subgroup of 11 women undergoing *in vitro* fertilization (IVF) protocols we obtained both GCs and blood cells in order to determine the existence of a possible correlation between ovarian and blood mtDNA content. Then, blood samples were obtained in three groups of women of comparable young age and one group of old women with physiological menopause (Table 1). The first group was constituted by 59 women with idiopathic POF,17 with primary and 42 with secondary amenorrhea and FSH serum levels exceeding 40 IU/L on at least two determinations [23]. The other 2 groups were selected among women undergoing ovarian hyperstimulation for IVF cycles. One group was constituted by poor responder women developing few co-dominant follicles and retrieving few (<5) oocytes despite elevated gonadotropin dosages (>300 IU per day) (PR; n = 42). The other was the group of control women, selected among those undergoing ovarian hyperstimulation using gonadotropin dosages ≤250 IU and retrieving ≥5 oocytes (Normal Responders, NR, n = 43). In both of these groups the main indication for IVF were male and tubal factors (88% in NR and 74% in PR). Finally another group is represented by very old control women with physiological menopause beyond 48 years of age (CPM, n = 53). Patients with ovarian cysts and/or those who were operated for ovarian cysts were excluded from all groups. All patients were of Caucasian origin.

**Table 1:** Anagraphical, clinical and biochemical parameters in the four groups of subjects.

Parameters	Group 1: POF (n = 59)	Group 2: PR (n = 42)(<5 eggs retrieved)	Group 3: NR (n = 43)(≥5 eggs retrieved)	Group 4: CPM (n = 53)
Age in years at blood samplingmean ±SE (range)	29.2±1.8* (15–41)	34.4±2.6 (27–39)	33±2.9 (27–38)	90±1.3 (82–105)
Age in years at menopausemean ±SE (range)	28.6±1.7 (14–39)	–	–	50.9±0.3 (48–58)
FSH (U/L) mean ±SE (range)	94.8±6.2* (30.2–160.0)	16.2±9.8 (5.1–53.0)	6.7±2.0 (1.9–12.7)	88.3±6.4 (69.0–105.2)
Ovarian volume (ml)	4.2±2.9*	5.4±2.8**	8.0±3.7	–
AFC^a^	0–1*	2.7±1.3**	7.3±2.7	–

a Antral Follicle Count per ovary.

* *p*<0.03 vs PR and NR.

** *p*<0.05 vs NR.

A blood sample was obtained from selected patients prior to the initiation of medical treatments (ovarian hyper-stimulation or hormone replacement therapy). Selection of patients with compromised ovarian reserve and controls was initially based on the outcome of previous ovarian hyperstimulation cycles, the antral follicle count (AFC on both ovaries <8) and the hormonal tests (day 3 serum FSH >12 U/L). The appropriateness of their inclusion was confirmed after the treatment cycle.

### MtDNA Determination in Circulating Blood Cells

Whole blood samples for the determination of mtDNA content were collected in EDTA-containing tubes. In these samples, the hemogram parameters including white blood cell (NR:6,074±1,352; PR:6,116±1,320; POF: 7,433±1,783; CPM: 5,917±1,441) and platelet (NR:212,040±56,350; PR:219,036±52,389; POF:211,571±41,279; CPM: 213,844±61,953) counts were similar in the different groups, a finding against the potential interference by significant variations in platelet count. The blood samples were immediately stored at −20°C and thawed simultaneously for mtDNA content determination. Total genomic DNA was isolated from whole-blood specimens by the Wizard Genomic DNA Purification Kit (Promega). MtDNA content was determined utilizing a quantitative real-time PCR (QPCR) by the Taqman method (Applied Biosystem 9700 HT Sequention Detection System) similarly to previously described protocols [24]. Specific probes were used to amplify a fragment of the mitochondrial D-loop region, Hs02596861_s1 MT-7S (Applied Biosystems), or a fragment of the nuclear genomic RNAse P, 4316844 RNAse P VIC (Applied Biosystems). PCR reactions were performed according to standard conditions for Taqman (Applied Biosystems): 50°C for 2′; 95°C for 10′; 40 cycles at 95°C 15′′, 60°C 1′. PCR assays were performed in triplicate for each DNA sample. Real-time PCR efficiencies were calculated from the given slopes in LightCycler software. The corresponding real-time PCR efficiencies (E) of one cycle in the exponential phase was calculated according to the equation: E = 10[−1/slope]. Investigated genes showed high real-time PCR efficiency rates; for D-Loop, 1.90 and RNAse P, 1.89 in the investigated range from 0.40 to 100 ng DNA input (n = 5) with high linearity (Pearson correlation coefficient r>0.98). We then determined the expression of mtDNA copy number relative to nuclear DNA using the efficiency correction method as described by Pfaffl et co [25]:


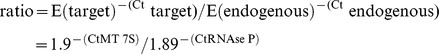


The primary endpoint of the study was the mtDNA content evaluation in women with intact and compromised ovarian reserve. Assumptions to determine the sample size included a type I and II error of 0.05 and 0.20, respectively.

### Analysis of Mitochondrial DNA Polymerase γ (POLG) Gene

*POLG* gene was analyzed by PCR amplification using intronic primers as previously described [26], and subsequently sequenced by automated nucleotide sequencing with the Big Dye terminator Ready Reaction Kit version 3.1 on a 3100 Genetic analyzer automatic sequencer (Applied Biosystem, CA, USA). The analysis was limited to the exons 7,8,17,18,21 on the basis of the reported mutations in the POLG gene in association with POI [19], [21], [27], [28].

## Results

**Figure 1:**
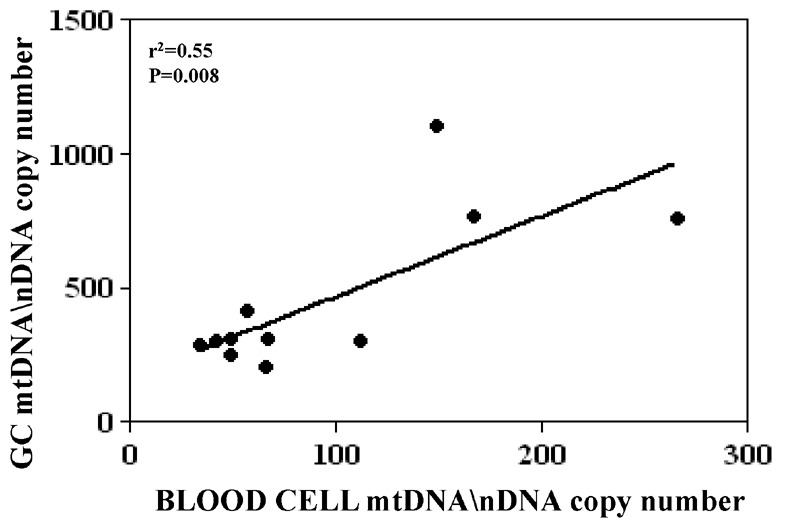
Correlation between peripheral blood and ovarian granulosa cell (GC) mitochondrial DNA content. Total DNA isolated from whole blood and GCs of a total of 11 women has been quantified by real-time quantitative PCR analysis using the RNAse P as an endogenous control. Data were analyzed by using comparative Ct method [25] and are expressed as relative quantification of mitochondrial on nuclear DNA copy numbers (mtDNA/nDNA). Regression analysis was obtained by GraphPad Prism 5.0.

In 11 women undergoing IVF protocols, the blood cell mtDNA/nDNA copy number correlated significantly (p = 0.008) with that of their GCs (Figure 1).

Age and BMI of NR, PR and POF women were comparable, though POF women tended to be younger (Table 1). CPM women were instead very old, 90±1.3 years, but had a BMI, 25±4 kg/ m^2^, similar to that of the other groups. Consistently with diagnosis, POF women had smaller ovaries without antral follicles in most cases. Antral follicle counts (AFC) and ovary volumes at ultrasound were lower in PR women, as compared to NR women.

**Figure 2:**
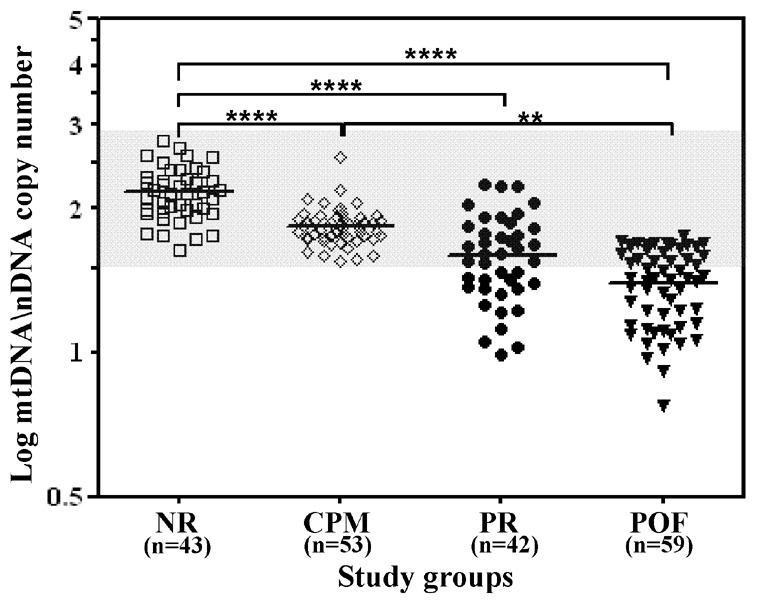
Relative quantification of mitochondrial on nuclear DNA copy number (mtDNA/nDNA) in peripheral blood cells. Total DNA isolated from whole blood has been quantified by real-time quantitative PCR analysis using the RNAse P as an endogenous control. The PCR data were analyzed by using a comparative Ct method [25] and values are here expressed as the Logarithm of mtDNA/nDNA copy number. Mean value for each group is graphically indicated by a line. The grey area is indicating the range observed in the NR group. The significant differences detected by one-way ANOVA test are indicated in the figure (****p<0.0001; **p<0.01).

Each DNA sample was run in at least three distinct Taqman assays in triplicate. Intra- and inter-assay coefficients of variability were <9.6% and <15.2%, respectively. Kurtosis and skewness indexes of the content in mtDNA were consistent with a normal distribution within the groups but variances differed. Data were thus compared using ANOVA test and Dunnett (T3) post-hoc test. The mean ±SE of the mtDNA/nDNA copy number was significantly lower in women with an impaired ovarian reserve (POF: 28.65±1.8; PR: 52.4±6.6; p<0.0001), in comparison to control women with an intact ovarian reserve (NR: 176.0±18.4) (Figure 2). In addition, the mtDNA/nDNA copy number was significantly higher in NR versus CPM (CPM: 69.6±3.1) (p<0.0001) and in CPM versus POF women (p<0.01). Instead, the Dunnett (T3) post-hoc test showed statistically significant differences for all comparisons, including PR versus POF (p = 0.005) or CPM versus PR (p = 0.014).

Considering the range of mtDNA/nDNA copy number observed in CPM women, lower values were found in 0/43 (0%) NR women, 19/42 (45%) PR and 38/59 (64%) POF. The analysis of *POLG* gene did not reveal any pathogenic variation in the 57 (38 POF and 19 PR) women with a mtDNA/nDNA copy number below the lower limit observed in the control groups.

## Discussion

In this study, we observed a significant correlation between ovarian and blood mtDNA content and then found a diminished mtDNA copy number in peripheral blood cells of women with an impaired ovarian reserve compared to controls. A biological grading clearly emerged showing the lowest levels in women with POF and the highest in those with conserved ovarian function. Interestingly, an intermediate level of mtDNA/nDNA copy number was found in women with a poor response to ovarian hyperstimulation (PR). The control groups of the present study consisted of women with a normal response to ovarian hyperstimulation (NR) and very old women with physiological menopause (CPM). Since aging is a condition associated with a progressive and generalized mitochondrial depletion [29]–[31], it is reasonable that mean mtDNA/nDNA copy number in old CPM women was found significantly lower than in young NR ones. However, the mtDNA amount of CPM women was always included within the range observed in NRs and significantly higher than that of young women with a premature impairment of their ovarian reserve. Therefore, a still undetermined mitochondrial DNA damage might represent a defect predisposing to premature ovarian insufficiency.

Since the reduction of the mtDNA/nDNA copy number was seen in the blood cells of subjects with compromised ovarian reserve and POF this defect appears as an intrinsic feature of the women. Though the defects of mitochondrial metabolism cause a wide range of human diseases affecting several tissues [32], the affected women in this study did not report any systemic manifestation at enrolment. One possible explanation for this incongruity is that the entity of the defect detected in this study is detrimental in the ovary while being tolerable for the other tissues. There is indeed evidence suggesting that biochemical dysfunction and consequent systemic manifestations occur only below a threshold level [32], [33] and ovarian tissue may be particularly susceptible to the risks associated with a relative mitochondrial DNA depletion. Consistently, folliculogenesis would require the bioenergetic support of a large number of mitochondria in both germinal and granulosa cells [16], [17]. Ultrastructural studies in human germinal cells showed that the number of mitochondria dramatically increases from about 10 in primordial germ cells to more than 100,000 in mature oocytes [34], [35]. This phenomenon correlating with a progressive increment of respiratory chain activity and oxygen consumption during follicle maturation [36]–[38]. In addition, Luoma et al. reported a relevant incidence of POF in women with *POLG* mutations, a protein essential for mtDNA replication [19]. Pagnamenta et al. recently confirmed this finding [21] and suggested to screen POF women for possible genetic mechanisms of mtDNA depletion even in the absence of evident manifestations such as the progressive external ophtalmoplegia (PEO). However, we could not find any pathogenic variations of *POLG* gene in POF or PR women with impaired mtDNA/nDNA copy number, thus suggesting that other mechanisms cause the mitochondrial DNA depletion in these women.

Based on these findings, POI might be interpreted as a condition of anticipated aging in which the ovarian defect may represent the first manifestation. In contrast, fertility beyond the age of 40 might be associated with longevity. Accordingly, centenarian women have been described to be four times more likely to have had children while in their forties than 73 year-old women [39]. This finding was recently confirmed by a comparative study of women from three large historical databases [40]. Interestingly, also the relatives of old mothers were shown to survive longer indicating the involvement of inheritable traits [41]. Our findings may then represent a suitable explanation for the correlation between late female fertility and longevity, and may also have implications regarding the theoretical basis of menopause and human lifespan. In line with this view stands the fact that a decreased fertility generally occurs before death in all female animals, and only human females go into menopause nowadays 30–50 years before death thanks to the recent progress of preventive medicine and therapeutics. These observations in humans are consistent with selection experiments in Drosophila in which the ability to produce eggs later in life is correlated with greater life expectancy [42].

Here, the difference in mtDNA content between POF/PR women and NR control women of similar age was highly significant with overlaps that were in several assays limited to about half of the cohorts. Since menopause has been repeatedly shown to arise earlier in women with a reduced responsiveness to ovarian hyperstimulation [43]–[45], blood cell mtDNA/nDNA copy number determination might represent a valuable test predicting the premature cessation of menses and ovarian failure in the occult phase of the disease, when the currently available tests are still not sufficiently efficient [1], [2], [4], [22].

In conclusion, the blood and ovarian mtDNA/nDNA copy numbers appear to be correlated and a diminished mtDNA content was found in peripheral blood cells of women with an impaired ovarian reserve. Therefore, POI may frequently occur as a consequence of a generalized and still undetermined mitochondrial defect in which the ovary appears as the first affected tissue. Finally, we propose the blood mtDNA/nDNA copy number determination as a promising tool for the prediction either of the POI risk or the poor response to ovarian hyperstimulation.
